# *Neisseria meningitidis* elicits a pro-inflammatory response involving IκBζ in a human blood-cerebrospinal fluid barrier model

**DOI:** 10.1186/s12974-014-0163-x

**Published:** 2014-09-13

**Authors:** Julia Borkowski, Li Li, Ulrike Steinmann, Natascha Quednau, Carolin Stump-Guthier, Christel Weiss, Peter Findeisen, Norbert Gretz, Hiroshi Ishikawa, Tobias Tenenbaum, Horst Schroten, Christian Schwerk

**Affiliations:** Department of Pediatrics, Pediatric Infectious Diseases, Medical Faculty Mannheim, Heidelberg University, Theodor-Kutzer-Ufer 1-3, 68167 Mannheim, Germany; Medical Research Center, Medical Faculty Mannheim, Heidelberg University, Theodor-Kutzer-Ufer 1-3, 68167 Mannheim, Germany; Institute of Medical Statistics and Biomathematics, Medical Faculty Mannheim, Heidelberg University, Ludolf-Krehl-Strasse 13-17, 68167 Mannheim, Germany; Institute for Clinical Chemistry, Medical Faculty Mannheim, Heidelberg University, Theodor-Kutzer-Ufer 1-3, 68167 Mannheim, Germany; Department of NDU Life Sciences, Nippon Dental University, School of Life Dentistry, Chiyoda-ku, Tokyo Japan

**Keywords:** Blood-cerebrospinal fluid barrier, Cellular immune response, Choroid plexus, Host-pathogen interactions, Microarray, *Neisseria meningitidis*, Toll-like receptors, Transcriptomics

## Abstract

**Background:**

The human-specific, Gram-negative bacterium *Neisseria meningitidis* (*Nm*) is a leading cause of bacterial meningitis worldwide. The blood-cerebrospinal fluid barrier (BCSFB), which is constituted by the epithelial cells of the choroid plexus (CP), has been suggested as one of the potential entry sites of *Nm* into the CSF and can contribute to the inflammatory response during infectious diseases of the brain. Toll-like receptors (TLRs) are involved in mediating signal transduction caused by the pathogens.

**Methods:**

Using a recently established *in vitro* model of the human BCSFB based on human malignant CP papilloma (HIBCPP) cells we investigated the cellular response of HIBCPP cells challenged with the meningitis-causing *Nm* strain, MC58, employing transcriptome and RT-PCR analysis, cytokine bead array, and enzyme-linked immunosorbent assay (ELISA). In comparison, we analyzed the answer to the closely related unencapsulated carrier isolate *Nm* α14. The presence of TLRs in HIBCPP and their role during signal transduction caused by *Nm* was studied by RT-PCR and the use of specific agonists and mutant bacteria.

**Results:**

We observed a stronger transcriptional response after infection with strain MC58, in particular with its capsule-deficient mutant MC58siaD^−^, which correlated with bacterial invasion levels. Expression evaluation and Gene Set Enrichment Analysis pointed to a NFκB-mediated pro-inflammatory immune response involving up-regulation of the transcription factor IκBζ. Infected cells secreted significant levels of pro-inflammatory chemokines and cytokines, including, among others, IL8, CXCL1-3, and the IκBζ target gene product IL6. The expression profile of pattern recognition receptors in HIBCPP cells and the response to specific agonists indicates that TLR2/TLR6, rather than TLR4 or TLR2/TLR1, is involved in the cellular reaction following *Nm* infection.

**Conclusions:**

Our data show that *Nm* can initiate a pro-inflammatory response in human CP epithelial cells probably involving TLR2/TLR6 signaling and the transcriptional regulator IκBζ.

**Electronic supplementary material:**

The online version of this article (doi:10.1186/s12974-014-0163-x) contains supplementary material, which is available to authorized users.

## Background

*Neisseria meningitidis* (*Nm*) is a human-specific Gram-negative bacterium that extracellularly colonizes the nasopharynx. Although *Nm* is often a non-pathogenic commensal, certain *Nm* strains have the potential to cause life threatening diseases, such as sepsis and meningitis, in susceptible individuals. In a first step, systemic invasion by crossing the mucosal epithelium leads to bacteremia in the host organism. Subsequently, to cause meningitis, the bacteria need to overcome the physiological barrier between the blood and the central nervous system (CNS) [1,2]. Structures known to separate the blood from the CNS are the blood–brain barrier and the blood-cerebrospinal fluid barrier (BCSFB). The bacteria may enter the subarachnoidal space by crossing the BCSFB of meningeal blood vessels, or they proceed into the ventricular system via the choroid plexus (CP) [3], where the morphological correlate of the BCSFB are the epithelial cells [4]. There is evidence that during an infection *Nm* interacts with the basolateral side of CP epithelial cells [5,6] and in an *in vitro* model of the BCSFB based on human choroid plexus papilloma (HIBCPP) cells *Neisseria* can enter and transmigrate across these CP epithelial cells by invasion from the physiologically relevant basolateral blood side [7]. An important virulence factor of *Nm* during the progress of meningitis is the bacterial capsule [8], and capsule-deficient mutants displayed higher invasion rates into HIBCPP cells *in vitro* [7].

The first line of host defense against invading pathogens is represented by receptors of the innate immune system belonging to the pattern recognition receptor (PRR) family. PRRs recognize evolutionary-conserved pathogen-associated molecular patterns (PAMPs) present on microorganisms. One central class of PRRs is represented by the Toll-like receptor (TLR) family, which are structurally characterized by a cytoplasmatic Toll/Interleukin-1 receptor (TIR) domain and extracellular leucine-rich repeats [9,10]. PAMPs recognized by TLRs include lipopolysaccharide (LPS) and lipooligosaccharide as well as lipoteichoic acid, which are components of bacterial cell walls, and which can be recognized by TLR4 and TLR2, respectively. Signaling by TLR2 can involve interactions with TLR1 or TLR6 for recognition of diacetylated (TLR2/TLR6) and triacetylated (TLR2/TLR1) lipopeptides [9]. Subsequent to recognition of PAMPs, TLRs recruit TIR-containing adaptor molecules, including MyD88 and TRIF, resulting in activation of the transcription factor NFκB, a process that requires the phosphorylation and degradation of inhibitory κB (IκB) proteins by kinases like IκB kinase α and β. Besides the typical IκB proteins, which mask the nuclear localization signal of NFκB and repress its nuclear translocation, the IκB family also contains members (IκBζ, Bcl-3, and IκBNS) that interact with NFκB in the nucleus and regulate transcription at the promoters of target genes [11,12].

Activation of NFκB signaling leads to the direct expression of early response genes, including genes encoding pro-inflammatory chemokines and cytokines like IL8, TNFα, IL1β, CXCL1, CXCL2, and CXCL3 (CXCL1–3 are also known as KC, MIP-2a, and MIP-2b, respectively), but also of additional genes involved in the NFκB-signaling such as the *nfkbiz* gene, which encodes the IκBζ protein. Association of IκBζ with the NFκB p50 subunit homodimer results in the recruitment of this transactivator complex to promoters with NFκB-binding sites [13] and the subsequent expression of several secondary response genes encoding for cytokines including IL6, a pro-inflammatory cytokine involved in the development of endotoxic shock [14], and others (IL12 p40, GM-CSF, G-CSF) [15]. Noteworthy, IL6 production is impaired in peritoneal macrophages from IκBζ knockout mice after stimulation of various TLRs, and IκBζ is also a key regulator of IL6 production in human monocytes, highlighting the role of IκBζ during inflammatory processes [15,16]. LPS, but also other TLR ligands (e.g., derived from *Legionella pneumophilia*) as well as IL1β have been shown to induce IκBζ [15,17-20].

Elevated cytokine levels in the cerebrospinal fluid (CSF) from patients suffering from bacterial meningitis have been shown in several studies. Cytokines and chemokines characteristically found in the CSF during bacterial meningitis pathogenesis include IL8, IL6, TNFα, CXCL1, IL1β, and MCP-1 as the most abundant [21–23]. It has been proposed that the CP contributes to the production of cytokines during inflammatory events in the CNS [24], and studies with primary porcine choroid plexus cells (PCPEC) have shown that infection with *Streptococcus suis* (*S. suis*) induces production of cytokines and chemokines including IL8, IL6, and TNFα [25]. These signaling molecules are known to act as chemoattractants leading to activation of leucocytes and their infiltration into the CNS potentiating a loss of barrier function and brain injury by generation of reactive oxygen metabolites, proteolytic enzymes, or toxic cytokines. The inflammatory host reaction therefore plays a crucial role for meningitis pathogenesis, which is rather the result of the inflammatory immune response of the host than of the presence of the pathogen itself [26-28]. A better understanding of the mechanisms involved would enable further therapeutic approaches.

Here, we investigate the cellular answer of HIBCPP cells to basolateral infection with *Nm in vitro* using microarrays and cytokine bead arrays. We show that *Nm* causes an inflammatory response characterized by the expression of cytokines and chemokines in concert with the transcriptional regulator IκBζ. Detailed analysis of TLR signaling furthermore reveals that induction of IκBζ and the immune response involves TLR2 rather than TLR4. Expression levels are most pronounced after infection with an acapsular mutant of a meningococcal disease isolate and correlate with the extent of bacterial invasion into HIBCPP cells.

## Methods

### Cell culture

The HIBCPP cell line and its use as a model of the human BCSFB have been described previously [7,29]. Briefly, HIBCPP cells were cultured in DMEM/F-12 (Ham) with 4 mM L-glutamine and 15 mM HEPES supplemented with 5 μg mL^−1^ insulin, 100 U mL^−1^ penicillin, and 100 μg mL^−1^ streptomycin as well as 15% heat inactivated fetal calf serum (FCS). For inverted cell culture insert based experiments 0.7 × 10^5^ cells were seeded on filter inserts (pore diameter 3.0 μm, pore density 2.0 × 10^6^ pores per cm^2^, growth area 0.33 cm^2^, from either Millipore, Schwalbach, Germany, or Greiner Bio-one, Frickenhausen, Germany) that were flipped over and placed in a medium flooded 12-well plate. Further cultivation and transepithelial electrical resistance (TEER) measurements were performed as previously described [7].

Isolation of human monocytes from healthy adult donors (approval was provided by the local ethics committee of the Medical Faculty of Mannheim, Heidelberg University (2009–327 N-MA)) by depletion of non-monocytes from peripheral blood mononuclear cells (PBMCs) was performed using Dynabeads for Untouched Human Monocytes (Invitrogen, Karlsruhe, Germany). PBMCs were isolated beforehand from fresh and non-coagulated potassium-EDTA blood by density sedimentation using Biocoll separation solution (Biochrom, Berlin, Germany) and LeucoSep Tubes (Greiner Bio-one) according to the manufacturer’s instructions.

### Bacterial strains

*Nm* strain MC58 (WUE2135) [30], the isogenic siaD mutant WUE2425 (MC58siaD^−^) [31] deficient for capsule production, the isogenic PorB mutant WUE4843 (MC58PorB^−^) deficient for the PorB protein, and the constitutively unencapsulated carrier isolate α14 [32,33] were stored at −80°C, plated on Chocolate Agar with Vitox (Oxoid, Wesel, Germany) and grown at 37°C in 5% CO_2_ atmosphere overnight. WUE4843 was constructed using chromosomal DNA of the *Nm* strain H44/76-Δcl3 [34]. All strains were kindly provided by H. Claus and U. Vogel (Institute for Hygiene and Microbiology, Würzburg, Germany). For determination of the lack of the outer membrane opacity protein Opc in strain α14 this publication made use of the Neisseria Multi Locus Sequence Typing website (http://pubmlst.org/neisseria/) developed by Keith Jolley and sited at the University of Oxford [35]. The development of this site has been funded by the Wellcome Trust and the European Union.

For infection assays few colonies from the overnight culture were subsequently cultured in Proteose Peptone Medium (PPM) supplemented with 0.042% NaHCO_3_, 0.01 M MgCl_2_, and 1% Polyvitex (bioMerieux, Lyon, France) to mid-logarithmic phase, washed with phenol red-free DMEM/F-12 with 4 mM L-glutamine and 15 mM HEPES supplemented with 5 μg mL^−1^ insulin and 1% FCS and diluted to an optical density at 600 nm (OD_600_) of 0.1.

For UV-inactivation, neisserial strains were grown in supplemented PPM and adjusted to an OD_600_ of 1.0. Inactivation was achieved by UV-irradiation of bacterial suspensions for 20 min in petri dishes with several rotations in between. Inactivated bacteria were frozen in −20°C until use. To determine the CFU/mL of the bacterial suspension, a serial dilution of an OD_600_ of 1.0 was plated on Chocolate Agar plates directly before irradiation. Inactivation was confirmed by plating and cultivation of undiluted inactivated bacterial suspension.

### Infection and stimulation of HIBCPP cells

Cells were seeded on inverted cell culture inserts and transferred to phenol red-free DMEM/F-12 with 4 mM L-glutamine and 15 mM HEPES supplemented with 5 μg mL^−1^ insulin and 1% FCS when TEER values reached 60 Ω × cm^2^. Infection from the basolateral side (mimicking the blood facing side *in vivo*) was carried out the next day when TEER values ranged around 500 Ω × cm^2^. Using the inverted cell culture insert system infection from the upper compartment simulates the pathophysiological situation *in vivo*, when pathogens invade the CSF from the blood. HIBCPP cells were infected with either strain MC58, MC58siaD^−^, or α14 at a multiplicity of infection of 10 at 37°C and 5% CO_2_ atmosphere for the indicated periods of time with antibiotic killing of the bacteria by addition of penicillin (100 U mL^−1^) and streptomycin (100 μg mL^−1^) after 4 h and prolonged infection up to 24 h.

Involvement of PRRs was investigated by stimulating HIBCPP cells from the basolateral side with “ultrapure” lipopolysaccharide from *E. coli* O111:B4 strain (LPS UP) as a TLR4 ligand, the synthetic diacylated lipopeptide (Fsl-1) as TLR2/TLR6 ligand, and the synthetic triacetylated lipopeptide PAM3CSK4 as TLR2/TLR1 ligand (all from InvivoGen, San Diego, CA, USA).

### Measurement of cell viability

Viability of HIBCPP cells was determined using a Life/Dead staining according to the manufacturer’s (Molecular Probes, Göttingen, Germany) instructions, where calcein stains viable cells in green and the non-membrane permeable ethidium bromide homodimer stains dead cells in red. The results were photodocumented by fluorescence microscopy.

### RNA extraction, quality control, cDNA preparation, and microarray performance

Following exposure to bacteria, cells were washed twice with PBS to exclude bacteria. Total RNA from HIBCPP cells was extracted using the QIAGEN RNeasy® Mini or Micro Kit (Qiagen, Hilden, Germany) according to the manufacturer’s instructions. During RNA purification an on-column DNAse digestion (RNase-Free DNase Set, Qiagen) was performed to avoid DNA carryover. RNA purity was evaluated by spectrophotometer (ND1000, Peqlab Biotechnoloy, Erlangen, Germany) and RNA quality of the microarray samples was additionally assessed using the Agilent RNA 6000 Nano Kit according to the manufacturer’s instructions and the Agilent Bioanalyzer 2100 (Agilent Technologies, Waldbronn, Germany). Samples with RNA integrity numbers higher than 9.8 were used for microarray analysis. After evaluation of the RNA samples, 100 ng of total RNA were reverse transcribed and synthesized into biotinylated aRNA using the 3′ IVT Expression Kit (Affymetrix, Santa Clara, CA, USA), 15 μg of purified and subsequently fragmented aRNA were hybridized with the array (GeneChip® Human Genome U133 Plus 2.0 Array, Affymetrix) for 16 h at 45°C and 60 rpm (GeneChip® Hybridisation Oven 640) with the help of the Hybridisation Wash and Stain Kit (Affymetrix). After washing the arrays were stained in the GeneChip® Fluidics Station 450 and data were documented using the GeneChip® Scanner 3000. Microarrays were performed in triplicate with three independent chips and samples from three independent experiments for every treatment. In each experiment, RNA from cells of three filter inserts was pooled.

### Microarray analysis

Fold changes of microarray data were calculated with the JMP Genomics from SAS software based on a mixed model ANOVA. Genes were regarded significantly up-regulated if differences in lg2 fold values ≥0.585 (=fold change ≥1.5) and down-regulated if lg2 fold values ≤ −0.585 (=fold change ≤0.67) with an additional criterion being that the corresponding *P* values were ≤0.001.

Determination of statistically overrepresented gene ontology (GO) terms was performed with the GOstat analysis tool [36]. GOstat was used with the following settings: GO gene-association database and commonly used gene collections, Affymetrix HG_U133_Plus_2; minimal length of considered GO paths, 5; maximal *P* value in GO output list, 1 × 10^−5^; cluster GOs, −1; and correct for multiple testing, false discovery rate (Benjamini).

Pathway analysis was done with the Gene Set Enrichment Analysis (GSEA) software developed by the Broad Institute of MIT and Harvard [37,38]. Molecular probe data were run against the gene set database of the C5 collection (GO gene sets) of the molecular signatures database (MSigDB) collection [39] to identify statistically enriched gene sets. Normalized Enrichment Scores were set ≥1.6 or ≤ −1.6 and *P* values ≥0.05, respectively.

### Reverse transcriptase polymerase chain reaction

Total RNA (500 ng), isolated and quantified as described above, was reverse transcribed using oligo dT primers included in the AffinityScript QPCR cDNA Synthesis Kit® (Agilent Technologies). PCR reactions were performed with the Taq PCR Core Kit (Qiagen) applying defined volumes of the generated cDNA and following the instructions provided by the manufacturer. PCR reaction mixtures were heated to 94°C for 2 min and were then subjected to the indicated cycles of denaturation (94°C, 30 sec), annealing (60°C, 30 sec), and extension (72°C, 2 min) followed by a final extension step at 72°C for 7 min. Subsequently, PCR products were visualized by agarose gel electrophoresis and ethidium bromide staining. Primers were designed using the following resources: PrimerBank [40,41], Primer3 software [42], RTPrimerDB [43], probe finder from the universal probe library from Roche Applied Science or as otherwise indicated. PCR primers used during this study are listed in Table 1.

**Table 1:** **Oligonucleotide primer**

**Gene symbol**	**Forward primer**	**Reverse primer**	**Size**	**Reference**
ACTNB	CATGTACGTTGCTATCCAGGC	CTCCTTAATGTCACGCACGAT	250	[44]
ADM	CGTCGGAGTTTCGAAAGAAG	CCCTGGAAGTTGTTCATGCT	232	[45]
CD14	ACTTGCACTTTCCAGCTTGC	GCCCAGTCCAGGATTGTCAG	202	This study
CXCL3	CGCCCAAACCGAAGTCATAG	GCTCCCCTTGTTCAGTATCTTTT	109	[44]
GAPDH	TGTTGCCATCAATGACCCCTT	CTCCACGACGTACTCAGCG	202	This study
IL6	AACCTGAACCTTCCAAAGATGG	TCTGGCTTGTTCCTCACTACT	159	This study
IL8	CAAGAGCCAGGAAGAAACCA	GTCCACTCTCAATCACTCTCAG	225	This study
MD2	GAAGCAGTATTGGGTCTGCAA	TTGGAAGATTCATGGTGTTGACA	209	This study
MyD88	CTGCTCGAGCTGCTTACCA	TAGCAGATGAAGGCATCGAA	236	This study
NFKBIZ	CAGTTCAAGTTAGCTGGCTGA	TCTGTGGAGAATACTGGTACAGG	177	This study
NOD1	ATCCTGGATGAATGCAAAGG	TCCTCCTTCTGTGGAGATGC	237	This study
NOD2	CTCCATGGCTAAGCTCCTTG	CCACACTGCCAATGTTGTTC	245	This study
TLR1	GCCTTGTCTATACACCAAGT	CCAATTGTTGCAGAGACTTC	310	[46]
TLR2	TCTCCCATTTCCGTCTTTTT	GGTCTTGGTGTTCATTATCTTC	125	[46]
TLR3	TAAACTGAACCATGCACTCT	TATGACGAAAGGCACCTATC	101	[46]
TLR4	TCCATAAAAGCCGAAAGG	CAGGGCTTTTCTGAGTCG	266	This study
TLR5	ACGGACTTGACAACCTCCAA	AGTGGATGAGGTTCGCTGTA	291	[46]
TLR6	CCCAAGGAGAAAAGCAAAC	TTCACCATCATCCAAGTAAAT	156	[46]
TLR7	CAGAGCTGAGATATTTGGACT	TTGTAAGTATCTGTTATCACCT	308	[46]
TLR8	CGGCAGAGTTATGCAAATAGT	GTAAGAGCACTAGCATTATCA	341	[46]
TLR9	GGCAAAGTGGGCGAGATGAG	AGTGGTGGTTGTCCCTGGTC	483	[46]
TLR10	CTCCCAACTTTGTCCAGAAT	TGGTGGGAATGCAATAGAAT	132	[46]
TNF	GAGCACTGAAAGCATGATCC	CGAGAAGATGATCTGACTGCC	234	This study
ZC3H12A	GGCAGTGAACTGGTTTCTGGA	GATCCCGTCAGACTCGTAGG	232	This study

ACTNB, β-actin; ADM, Adrenomedullin; CD, Cluster of differentiation; CXCL, Chemokine (C-X-C motif) ligand; GAPDH, Glyceraldehyde-3-phosphate dehydrogenase; IL, Interleukin; MyD, Myeloid differentiation; NFKBIZ, NF-kappa-B inhibitor zeta; NOD, Nucleotide-binding oligomerization domain; TLR, Toll-like receptor; TNF, Tumor necrosis factor α; *ZC3H12A*, Zinc finger CCCH-type containing 12A.

### Quantitative real-time PCR analysis (qPCR)

cDNA was prepared as described above. Subsequent quantitative real-time polymerase chain reaction (qPCR) was performed using the Brilliant II SYBR Green QPCR Master Mix (Agilent Technologies) according to the manufacturer’s instructions with initial denaturation (95°C, 10 min) followed by 40 cycles of denaturation (95°C, 30 sec), annealing (60°C, 60 sec), extension (72°C, 60 sec), and a denaturation curve (95°C, 60 sec; 60°C, 30 sec; 95°C, 30 sec).

qPCR data analysis was carried out according to a genorm-based approach [47] with normalization to β-actin and GAPDH as control genes in duplicates and triplicates, respectively. For each gene of each sample the quantity and standard deviation was calculated. The efficiency of amplification for each primer (ACTNB (β-actin), GAPDH, CXCL3, IL8, IL6, NFKBIZ, TNF, and ADM) was determined. The data were standardized to a normalization factor representing the geo-mean of the quantity of the control genes β-actin and GAPDH to get the relative expression values. Analysis of expression levels was done by normalizing relative expression values to the mean of the untreated control after 4 h and after 0 h for samples analyzed by microarray and for kinetic induction of *nfkbiz* and *il6*, respectively. Subsequently, the mean and standard deviation for every treatment were calculated for absolute fold-increase values.

### Cytokine and chemokine secretion in HIBCPP cell supernatants

Supernatants were collected after infection with *Nm* strains. After 4 h, bacteria were inactivated by addition of penicillin (100 U mL^−1^) and streptomycin (100 μg mL^−1^) to the indicated final concentrations and supernatants were taken after the indicated incubation periods.

To determine the response of infected HIBCPP cells, supernatants were analyzed using the Luminex array technology. A commercially available multiplex cytokine bead array for the detection of 42 different cytokines and chemokines (Millipore, Milliplex® Human Cytokine/Chemokine Kit, MPXHCYTO60KPMX42) was used to calculate IL6, G-CSF, GM-CSF, and TNFα levels as well as IL8 and panGro/CXCL1–3 levels. Secreted levels of IL8 and panGro/CXCL1–3 were analyzed diluted in a selected duplex array.

Sample volumes of 25 μL were used diluted or undiluted and the kit was run according to the manufacturer’s instructions. For all conditions, two samples out of a single experiment were measured with single value measuring. Mean values and standard deviation were calculated from those two samples. Standard curves including all cytokines (in duplicates) were generated using the reference cytokine concentrations supplied. All incubation steps were performed at room temperature and in the dark to protect the beads from light. Cytokine concentrations were read on the Luminex 100™ system (Luminex, Austin, TX, USA). The detection limit for any analyte was 3.2 pg mL^−1^ with a dynamic range up to 10,000 pg mL^−1^ according to the manufacturer’s instructions.

Additionally, the concentration of IL6 in cell culture supernatants was quantified by ELISA (Human IL-6 High Sensitivity ELISA KIT, Cell Sciences® Inc., Canton, MA, USA) according to the manufacturer’s instructions. Two identically treated samples from one experiment were pooled and 100 μL of the pooled samples were used to determine IL6 concentrations. The sensitivity limit was 0.81 pg mL^−1^ of IL6. Standard curves were generated up to 50 pg mL^−1^ with the IL6 standard supplied in the kit. To calculate IL6 concentrations elongation of the standard curve had to be done as concentrations were slightly above the detection limit.

### Immunoblot

Following bacterial infection or stimulation with TLR ligands cells were washed with PBS. Whole protein was extracted with modified RIPA buffer (Millipore, Billerica, MA, USA) containing 1-fold Protease inhibitor cocktail and 1 mM Na_3_VO_4_. Subsequently, lysates were centrifuged for 10 min at 18,000 × *g*. Whole protein content was determined with the Lowry method (DC Protein Assay, BioRad, München, Germany) according to the manufacturer’s instructions. Protein samples were spiked with loading buffer and sample reducing agent (both Invitrogen) and equal amounts of protein were subjected to electrophoresis (MOPS running buffer, Invitrogen, 200 V). Proteins were separated on Bis Tris NuPage® gels (Invitrogen) and transferred onto nitrocellulose membranes using standard conditions. The primary antibodies recognizing IκBζ (1:1,000 dilution) and β-actin (Sigma-Aldrich, Steinheim, Germany; 1:10,000 dilution), respectively, were detected using anti-rabbit or anti-mouse HRP-conjugated secondary antibodies (both from Millipore, Temecula, CA, USA; 1:5,000 dilution) and the appropriate substrate (Immobilon Western Kit; Millipore, Billerica, MA, USA).

### Determination of bacterial invasion by double immunofluorescence

Invasion was determined as previously reported [48] with some modifications as described [7]. Noteworthy, after 4 h of infection, extracellular *Nm* including the carrier isolate α14 were detected with the primary antibody anti-*Nm* α-OMP (1:200). Formaldehyde fixation and incubation with a secondary antibody (Alexa Fluor 594 (red) chicken anti-rabbit, 1:500; Molecular Probes, Oregon, USA) ensure subsequent visualization. Permeabilization with PBS/0.5% Triton X-100/1% bovine serum albumin allows access to the invaded intracellular *Nm*, which are detected in a second incubation step with the anti-*Nm* α-OMP (1:200) antibody. Finally, intra- and extracellular bacteria were stained by incubation with a secondary antibody (Alexa Fluor 488 (green) chicken anti-rabbit antibody, 1:500; Molecular Probes). To stain the actin cytoskeleton and cell nuclei, respectively, the antibody dilution contains, in parallel, Phalloidin Alexa Fluor 660 (Molecular Probes) and 4′,6-diamidino-2-phenylindole dihydrochloride (DAPI; Calbiochem, Darmstadt, Germany) (1:50,000). Embedding of the isolated filter membranes in ProLongAntifadeReagent (Invitrogen) enables examination. Images were acquired with Zeiss Apotome and Axiovision software (Carl Zeiss, Jena, Germany) using a 636/1.4 objective lens. The image acquisition was carried out using the Zeiss scanning software Axiovison 4.6 and Axiovison module Inside 4D. To determine invasion rates, 20 fields of view (3,626 μm^2^) were counted for intracellular *Nm* (green) and calculated for the whole filter area. Numbers of intracellular *Nm* were set in relation to the growth of each strain determined in parallel. Assays were performed at least in triplicate for each condition and repeated at least three times.

### Statistical analysis

Statistical analysis for invasion of *Nm* into HIBCPP cells was done using Student’s *t*-test after testing for normal distribution and differences of variances. *P* values were considered significant, highly significant, or extremely significant, when <0.05, <0.01, or <0.001, respectively. Data represent means ± standard deviation.

Statistical calculations for qPCR and ELISA analyses were performed with the SAS system, release 9.3 (SAS Institute Inc., Cary, NC, USA). Quantitative parameters are presented as mean values and standard deviations. For normally distributed data a one-way analysis of variance (ANOVA) was performed to compare the mean values of differently treated cells. Adjustment for multiple comparisons was done by Tukey test. Test results with *P* <0.05 are considered statistically significant; *P* <0.01, very significant; and *P* <0.0001, highly significant.

### Microarray data accession number

All microarray data described in this study have been submitted to Gene Expression Omnibus (http://www.ncbi.nlm.nih.gov/projects/geo/) under accession number GSE42870.

## Results

### A neisserial carrier isolate invades HIBCPP cells only marginally from the basolateral side

**Figure 1:**
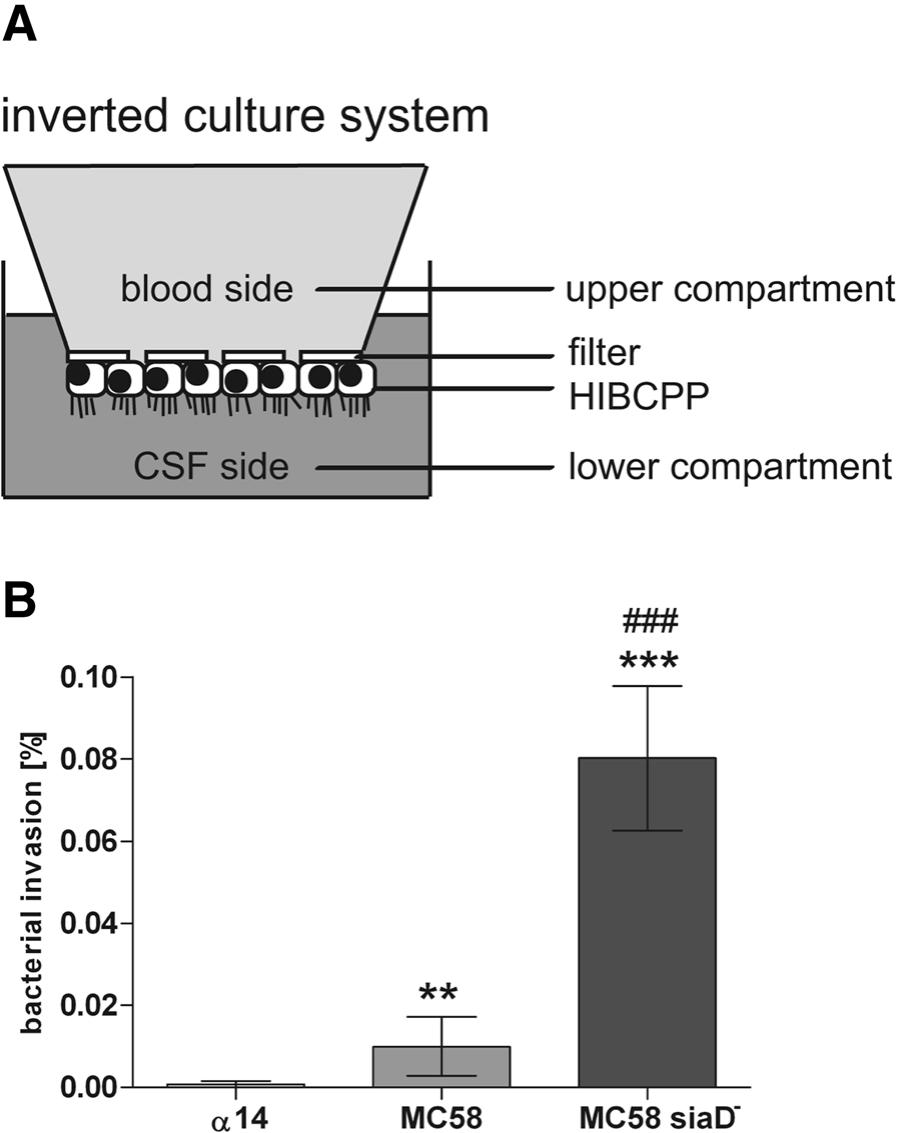
**Invasion of** ***Nm*** **into HIBCPP cells.** Invasion assays were performed in the inverted cell culture insert system. Invasion of the different *Nm* strains was analyzed by double immunofluorescence. **(A)** Schematic representation of the inverted cell culture insert system. Cells are grown on the lower side of the filter supports. Microvilli are indicated at the apical side. **(B)** HIBCPP cells were infected for 4 h at a multiplicity of infection of 10 and subsequently subjected to double immunofluorescence staining to distinguish intracellular and extracellular bacteria. Basolateral invasion was observed for *Nm* MC58 and the acapsular mutant strain MC58siaD^−^. Invasion was significantly attenuated by the presence of a capsule. Only marginal invasion was detected after infection with the carrier isolate α14. ** (highly significant; *P* <0.01), *** (extremely significant; *P* <0.001); when MC58 or MC58siaD^−^, respectively, were compared to α14. ### (extremely significant; *P* <0.001); when MC58siaD^−^ was compared to MC58.

We have previously shown that the *Nm* strain MC58, a disease isolate, invades HIBCPP cells polar from the physiologically-relevant basolateral side with the capsule attenuating invasion properties [7]. Using HIBCPP cells grown in an inverted cell culture insert system (Figure 1A) we now compared basolateral invasion of the MC58 strain and its acapsular mutant MC58siaD^−^ with that of the non-capsulated, commensal carrier isolate α14. Invasion was performed for 4 h, since we have previously seen that MC58 and MC58siaD^−^ invade HIBCPP cells within that time frame [7]. Analysis by double immunofluorescence microscopy confirmed stronger invasion of the non-encapsulated strain MC58siaD^−^ compared to its isogenic wild type. In contrast, only marginal invasion could be observed for the carrier isolate α14 (Figure 1B). Treatment of HIBCPP cells with bacteria did not lead to a significant impairment of cell viability as determined by a Life/Dead assay (data not shown).

### Microarray analyses reveal induction of pathways involving NFκB and IκBζ in *Nm* infected HIBCPP cells

We were interested in characterizing the transcriptional response of HIBCPP cells to infection with *Nm* from the basolateral side. For this purpose we treated HIBCPP cells grown in the inverted cell culture insert system with the *Nm* strains α14, MC58, and MC58siaD^−^. We analyzed HIBCPP cells infected with *Nm* for 4 h, since at this time point we had confirmed specific invasion of *Nm* MC58 and MC58siaD^−^ into HIBCPP cells (Figure 1B). To determine gene expression levels we used commercially available Gene Expression Chips covering over 47,000 transcripts.

**Figure 2:**
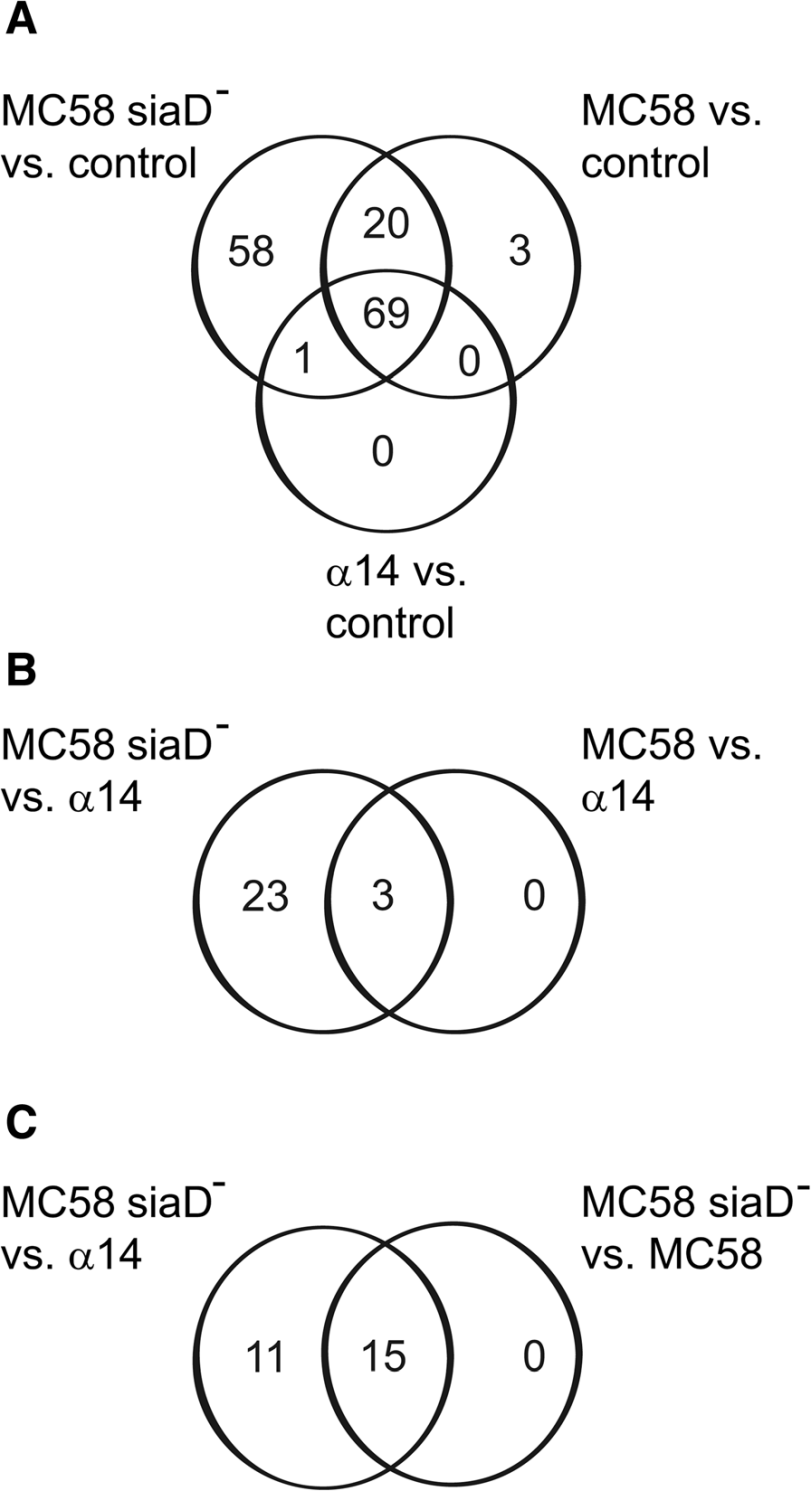
**Schematic representation of the numbers of genes found regulated during microarray analyses in HIBCPP cells after infection with** ***Nm*** **.** Regulated genes were identified by comparison of cells infected with *Nm* strain α14, MC58, or MC58siaD^−^ against the uninfected control **(A)**, by comparison of cells infected with *Nm* strain MC58 or MC58siaD^−^ against cells infected with *Nm* α14 **(B)**, or by comparison of cells infected with strain MC58siaD^−^ against strain α14 or against strain MC58 **(C)**, respectively.

We first compared the expression levels of HIBCPP cells treated with *Nm* with uninfected control cells. Genes were considered up- or down-regulated when changes in expression were ≥1.5-fold or ≤0.67-fold, respectively, and corresponding *P* values were ≤0.001. The results of these comparisons are schematically depicted in Figure 2A. Infection of HIBCPP cells with the carrier isolate α14 lead to the modulation of 70 genes (68 up-regulated, 2 down-regulated). When HIBCPP cells were treated with the MC58 strain, 92 genes were regulated (88 up-regulated, 4 down-regulated). These genes contained all but one of the 70 genes with changed expression levels after treatment with *Nm* α14 and 23 additional genes, which were not modulated by α14. Gene expression analysis of HIBCPP cells infected with the acapsular mutant strain MC58siaD^−^ revealed regulation of 148 genes (137 up-regulated, 11 down-regulated). These genes contained all 70 genes modulated by α14 and 89 of the 92 genes regulated by the MC58 wild type. The expression of 58 additional genes in HIBCPP cells was modified by MC58siaD^−^, which were not influenced by the other two strains.

The genes identified to be regulated by *Nm* during the microarray analyses are summarized in Additional file 1: Table S1. Noteworthy, these included various genes involved in the innate immune response and inflammation (*ccl20*, *cxcl6*, *cxcl5*, *cxcl3*, *cxcl2*, *cxcl8*, *tnf*, *il23a*, *ptgs2*, *lif*, *ltb*, *f3*, *tnfaip2*, *tnaip3*, *plau*, *plaur*) as well as genes participating in the regulation of NFκB-signaling (*bcl3*, *bcl10*, *nfkbia*, *nfkibz*, *nfkbie*, *birc3*). Further regulated genes encoded for intercellular adhesion molecules (*icam1*), negative feedback regulators of inflammatory and apoptotic pathways, apoptotic proteins, signaling molecules of the Ephrin pathway, molecules involved in MAP-Kinase signaling, vasoconstrictory peptides, several transporters, and multiple proteins playing a role in transcriptional regulation. We also identified genes involved in oxidative stress response or acting as hypoxic sensors as well as genes regulating the actin cytoskeleton and those which are responsible for processing of mRNAs like RNAses (*zc3h12a*). Additionally, *Neisseria* regulated genes coding for proteins participating in the carbohydrate metabolism (Additional file 1: Table S1).

In the next step we were interested to identify the genes significantly regulated in HIBCPP cells by strains MC58 and MC58siaD^−^ when compared to the carrier isolate α14. During this analysis we found only 3 genes which were significantly up-regulated by the wild type strain MC58. In contrast, expression of 26 genes was significantly increased by the acapsular mutant MC58siaD^−^, which contained the 3 genes regulated by the wild type (Figure 2B). Of these 26 genes, 15 were significantly up-regulated in HIBCPP cells by MC58siaD^−^ when setting the wild type MC58 as control (Figure 2C).

The genes up-regulated by MC58 or MC58siaD^−^, respectively, when compared to α14 are listed in Table 2. Noteworthy, the 26 genes, which are significantly up-regulated by MC58siaD^−^ with α14 set as control, contained several genes encoding for proteins involved in the immune response, including those encoding for the chemokines CXCL2 and CXCL3 as well the gene *nfkbiz* encoding for IκBζ, which were up-regulated by both MC58 and MC58siaD^−^. To gain information regarding the biological function of the genes listed in Table 2, we employed the GOstat software tool [36] to identify statistically over-represented GO terms. The overrepresented GO terms identified by analyzing genes found to be induced by MC58siaD^−^ with α14 or MC48 set as control are listed in Tables 3 and 4, respectively. These GO terms strongly corroborate the regulation of genes specifically participating in an inflammatory cellular response involving cytokine and chemokine activity.

**Table 2:** **Genes significantly more strongly regulated by the capsule-deficient mutant strain MC58siaD** ^**−**^ **compared with MC58 and the carrier isolate α14 as well as genes identified to be significantly more strongly regulated comparing expression levels of cells infected with MC58 and the carrier isolate α14 with FC ≥1.5 FC ≤0.67 and a corresponding** ***P*** **value ≤0.001**

**UniGene_ID**	**Gene title**	**Gene symbol**	**Fold change**		
			**MC58siaD** ^**−**^ **vs. MC58**	**MC58siaD** ^**−**^ **vs. α14**	**MC58 vs. α14**
Hs.75765	chemokine (C-X-C motif) ligand 2	CXCL2	2.25	4.03	1.79
Hs.75498	chemokine (C-C motif) ligand 20	CCL20	2.33	3.45	
Hs.89690	chemokine (C-X-C motif) ligand 3	CXCL3	1.94	3.28	1.69
Hs.319171	Nuclear factor of kappa light polypeptide gene enhancer in B-cells inhibitor, zeta	NFKBIZ	1.77	3.01	1.70
Hs.624	interleukin 8	IL8	1.77	2.51	
Hs.196384	prostaglandin-endoperoxide synthase (prostaglandin G/H synthase and cyclooxygenase)	PTGS2	1.99	2.47	
Hs.643447	intercellular adhesion molecule 1	ICAM1	1.80	2.35	
Hs.211600	tumor necrosis factor, alpha-induced protein 3	TNFAIP3	1.61	2.16	
Hs.81328	nuclear factor of kappa light polypeptide gene enhancer in B-cells inhibitor, alpha	NFKBIA	1.55	2.14	
Hs.241570	tumor necrosis factor (TNF superfamily, member 2)	TNF	1.54	2.14	
Hs.376208	Lymphotoxin beta (TNF superfamily, member 3)	LTB	1.70	2.13	
Hs.164021	chemokine (C-X-C motif) ligand 6 (granulocyte chemotactic protein 2)	CXCL6	1.92	2.08	
Hs.77274	plasminogen activator, urokinase	PLAU	1.70	1.98	
Hs.656294	zinc finger CCCH-type containing 12A	ZC3H12A		1.93	
Hs.432132	G0/G1switch 2	G0S2	1.56	1.86	
Hs.522109	solute carrier family 6 (amino acid transporter), member 14	SLC6A14	1.52	1.73	
Hs.525607	tumor necrosis factor, alpha-induced protein 2	TNFAIP2		1.67	
Hs.436061	interferon regulatory factor 1	IRF1		1.63	
Hs.591849	chromosome 8 open reading frame 4	C8orf4		1.62	
Hs.515415	inositol 1,4,5-trisphosphate 3-kinase C	ITPKC		1.62	
Hs.127799	baculoviral IAP repeat-containing 3	BIRC3		1.61	
Hs.124940	Rho family GTPase 1	RND1		1.60	
Hs. 98309	interleukin 23, alpha subunit p19	IL23A		1.56	
Hs.115263	epiregulin	EREG		1.55	
Hs.632267	syndecan 4	SDC4		1.55	
Hs.656630	230333_at	---		1.53

**Table 3:** **Statistically overrepresented GO terms of genes, which are significantly more strongly regulated by the capsule-deficient mutant strain MC58siaD** ^**−**^ **compared with the carrier isolate α14**

**GO term**	**Count**	**Total**	***P*** **value**
Cytokine activity	8	215	2.95 × 10^−07^
Response to wounding	9	377	4.97 × 10^−07^
Chemokine activity	5	46	9.51 × 10^−07^
Chemokine receptor binding	5	47	9.51 × 10^−07^
Taxis	6	133	3.09 × 10^−06^
Chemotaxis	6	133	3.09 × 10^−06^
G-protein-coupled receptor binding	5	69	3.90 × 10^−06^
Inflammatory response	7	268	6.08 × 10^−06^

Count, number of genes associated with given GO term; Total, number of genes in analysis associated with given GO term. *P* values were corrected according to Benjamini and Hochberg as implemented in GOstat.

**Table 4:** **Statistically overrepresented GO terms of genes, which are significantly more strongly regulated by the capsule-deficient mutant strain MC58siaD** ^**−**^ **compared with the wild type strain MC58**

**GO term**	**Count**	**Total**	***P*** **value**
Cytokine activity	7	215	5.66 × 10^−08^
Chemokine activity	5	46	5.66 × 10^−08^
Chemokine receptor binding	5	47	5.66 × 10^−08^
Taxis	6	133	8.64 × 10^−08^
Chemotaxis	6	133	8.64 × 10^−08^
G-protein-coupled receptor binding	5	69	2.05 × 10^−07^
Response to wounding	7	377	8.74 × 10^−07^
Inflammatory response	7	268	3.60 × 10^−06^

Count, number of genes associated with given GO term; Total, number of genes in analysis associated with given GO term. *P* values were corrected according to Benjamini and Hochberg as implemented in GOstat.

For interpretation of our expression data we performed, in a second step, a GSEA to evaluate the data with respect to known biological mechanisms and classification into gene sets [37]. With this method, we identified three gene sets that were significantly overrepresented by the strains MC58 and MC58siaD^−^ (Table 5). Noteworthy, those gene sets, namely “cytokine metabolic process”, “positive regulation of translation”, and “regulation of cytokine biosynthetic process”, included the genes *ltb*, *epr*, *bcl10*, *bcl3*, *il6*, and *il12b* that were commonly enriched in all three gene sets.

**Table 5:** **Gene sets identified by Gene Set Enrichment Analysis (GSEA) and significantly regulated by strains MC58 and MC58siaD** ^**−**^ **, respectively, compared with the carrier isolate α14**

**Gene set**	**MC58 vs. α14**		**MC58siaD** ^**−**^ **vs. α14**	
	**NES**	**NP**	**NES**	**NP**
Cytokine metabolic process	1.60	0.005	1.73	0.000
Positive regulation of translation	1.62	0.007	1.75	0.000
Regulation of cytokine biosynthetic process	1.65	0.007	1.77	0.000

NES, Normalized enrichment score; NP, Normalized *P* value.

### HIBCPP cells produce cytokines and chemokines most prominently after infection with an acapsular mutant of a neisserial disease isolate

**Figure 3:**
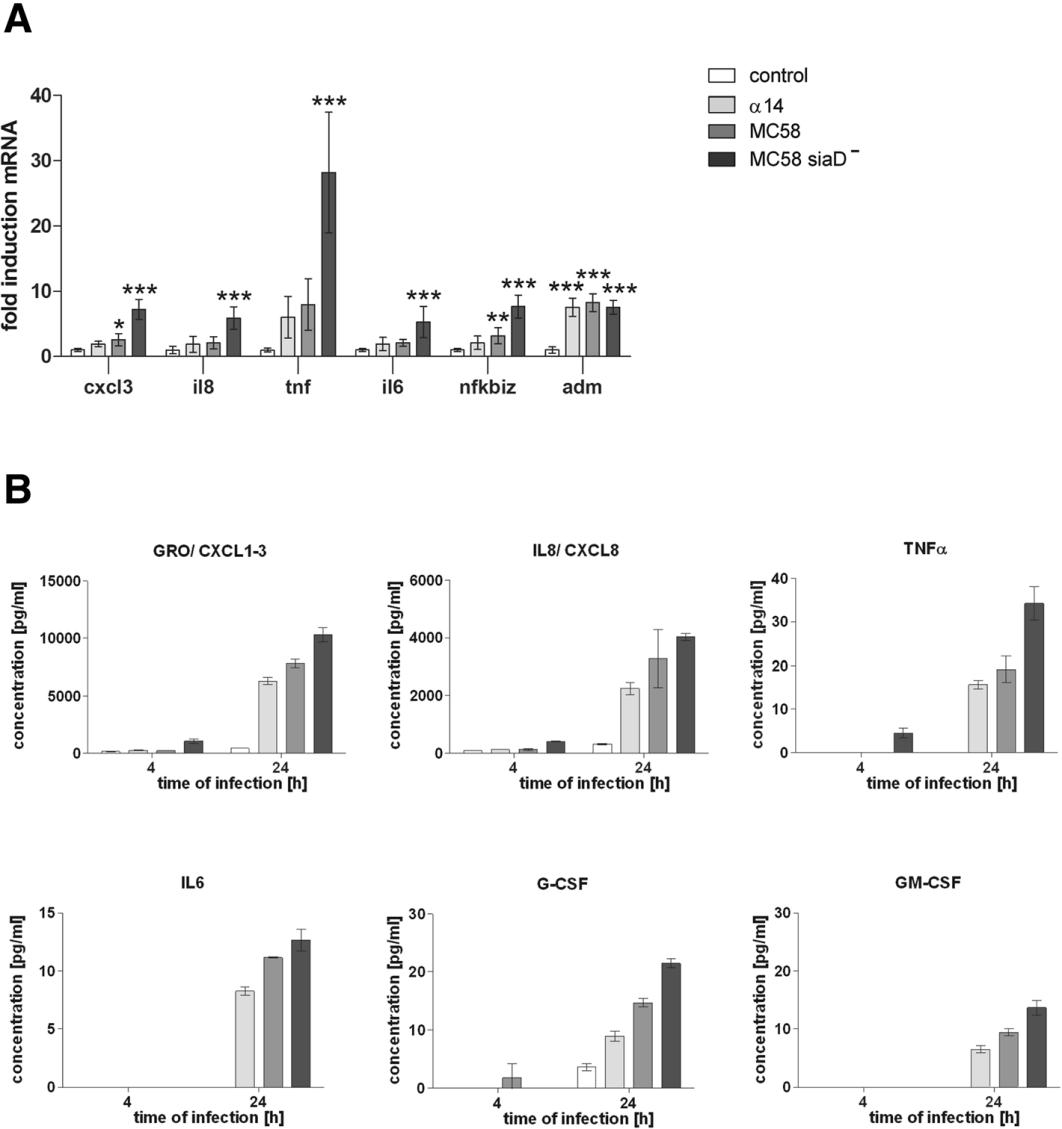
**qPCR analysis of selected genes and** ***Nm*** **-induced cytokine production in infected HIBCPP cells. (A)** Expression of genes coding for IκBζ, IL6, CXCL1-3, IL8, TNFα, and ADM was quantified by qPCR and a genorm-based approach with simultaneous normalization to β-actin and GAPDH (n = 6). * (significant; *P* <0.05), ** (highly significant; *P* <0.01), *** (extremely significant; *P* <0.001); when cells treated with α14, MC58, or MC58siaD^−^, respectively, were compared to the uninfected control. **(B)** Concentrations of IL6, G-CSF, GM-CSF, CXCL1-3, IL8, and TNFα were measured in HIBCPP cell culture supernatants after infection. In all experiments, cells were treated for 4 h with bacteria followed by antibiotic killing of the bacteria and further incubation up to 24 h. Bacteria were applied at a multiplicity of infection of 10, untreated cells served as a control.

In the following experiments, we were interested in investigating the relevance of up-regulation of cytokine/chemokine and IκBζ gene expression in more detail. For this purpose we first confirmed the expression of selected genes in HIBCPP cells after stimulation with *Nm* by quantitative real-time PCR (qPCR). As can be seen in Figure 3A, similarly to the results obtained during the microarray analyses (Additional file 1: Table S1), expression of the genes *cxcl3*, *il8*, *tnf*, and *nfkbiz* (encoding for IκBζ) was elevated after neisserial infection with the strongest effect caused by the MC58siaD^−^ strain (α14 < MC58 < MC58siadD^−^). The IκBζ target gene *il6* was also induced by *Nm*, again most pronounced by MC58siaD^−^. In contrast, the gene encoding for adrenomedullin (*adm*) was activated to the same extent by all three *Nm* strains, a result that was also observed during the microarray analyses (Additional file 1: Table S1). Treatment of HIBCPP cells with UV-inactivated *Nm* still lead to the induction of *nfkbiz*, *il6*, *il8*, and *zc3h12a* by all three strains (Additional file 2: Figure S1).

The strong overrepresentation of genes involved in cytokine and chemokine activity identified during the microarray analysis prompted us to investigate the levels of cytokine and chemokine release after infection with *Nm* using a cytokine bead array. Protein levels of a cytokine/chemokine panel were determined in the supernatants of uninfected control cells as well as after treatment of HIBCPP cells with the three *Nm* strains for different time frames as described in Methods. Viability of HIBCPP cells under these conditions was confirmed by a Life/Dead Assay (data not shown). The levels of selected cytokines and chemokines after 4 h and 24 h are shown in Figure 3B. Indeed, protein levels of cytokines and chemokines were up-regulated after infection with *Nm* with the most pronounced effect caused by MC58siaD^−^. Among the proteins analyzed, the most abundantly produced were IL8 and panGRO/CXCL1–3, corroborating the results of the microarray analyses. Increased production of TNFα and IL6, further notable signaling molecules in the beginning of inflammation [49], could also be detected. In addition to IL6, secretion of two other proteins encoded by IκBζ target genes, G-CSF and GM-CSF, was elevated after infection of HIBCPP cells. Subsequently to the 24 h time-point, levels of cytokines or chemokines analyzed in the supernatants of the HIBCPP cells were not or only marginally increased (data not shown).

### Kinetic profile of IκBζ and IL6 expression in *Neisseria*-infected HIBCPP cells

The *nfkbiz*-encoded IκBζ is an inducible nuclear IκB protein required for subsequent expression of several target genes including *il6* [15,19]. The *il6* gene was identified during the GSEA analysis, and IL6 is produced by HIBCPP cells after infection with *Nm*, most pronounced by MC58siaD^−^. In order to analyze the temporal regulation of *nfkbiz* and *il6* after neisserial infection, HIBCPP cells were treated with the three *Nm* strains for up to 24 h as described in Methods and gene expression was investigated by qPCR.

**Figure 4:**
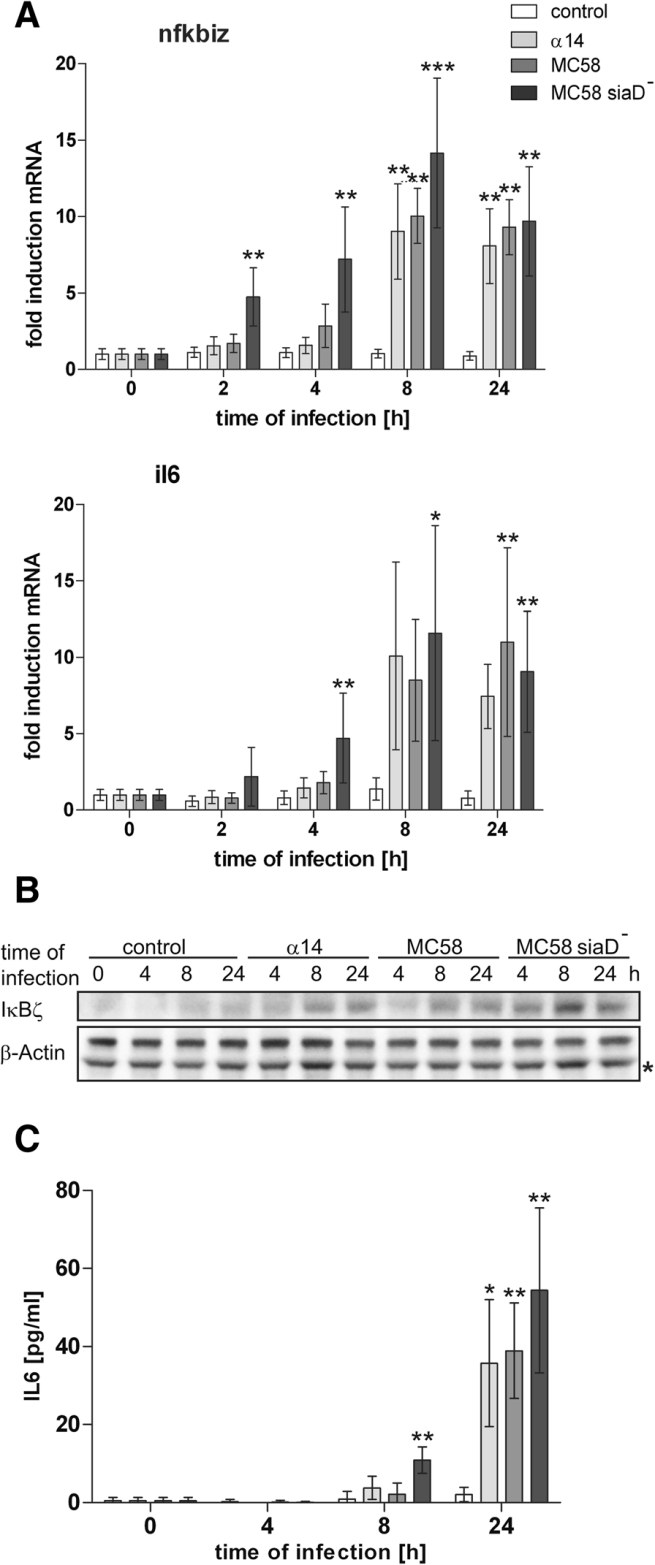
**Kinetic expression profile of IκBζ and IL6 was determined in infected HIBCPP cells. (A)** HIBCPP cells were challenged with *Nm* strains α14, MC58, or MC58siaD^−^ for the indicated time as described in Methods. Uninfected control cells served as control. Expression of IκBζ and IL6 was analyzed by qPCR and a genorm-based approach with simultaneous normalization to β-actin and GAPDH (n = 5). **(B)** Expression of IκBζ protein was analyzed by immunoblot analysis. Detection of β-actin protein levels served as control. A putative unspecific band is labelled with an asterisk (*). **(C)** IL6 expression was analyzed by ELISA (n = 5). For all experiments cells were treated for 4 h with bacteria followed by antibiotic killing of the bacteria and further incubation for the indicated time points. Bacteria were applied at a multiplicity of infection of 10, untreated cells served as a control. * (significant; *P* <0.05), ** (highly significant; *P* <0.01), *** (extremely significant; *P* <0.001); when cells treated with α14, MC58, or MC58siaD^−^, respectively, were compared to the uninfected control.

Expression of *nfkbiz* was induced rapidly following infection with *Nm*. After 2 h of infection with the mutant strain MC58siaD^−^, *nfkbiz* was significantly up-regulated ~5-fold as compared to uninfected control cells and maximum levels were detected 8 h post infection (Figure 4A). Consistent with mRNA levels, we detected IκBζ protein expression after 4 h of infection by immunoblot analysis (Figure 4B). In contrast, induction of *il6* by MC58siaD^−^ was non-significant after 2 h. A significant induction of *il6* after challenge with MC58siaD^−^ was observed firstly after 4 h and secretion of IL6 protein was detectable even later after 8 h of infection (Figure 4C).

The two other strains, MC58 and α14, induced a delayed expression of IκBζ and IL6. At later time points, however, induction levels comparable to those for the mutant strain were also observed for MC58 and α14 (Figure 4A–C).

### TLR2/TLR6 induction contributes to inflammatory response gene activation in *Nm*-infected HIBCPP cells

**Figure 5:**
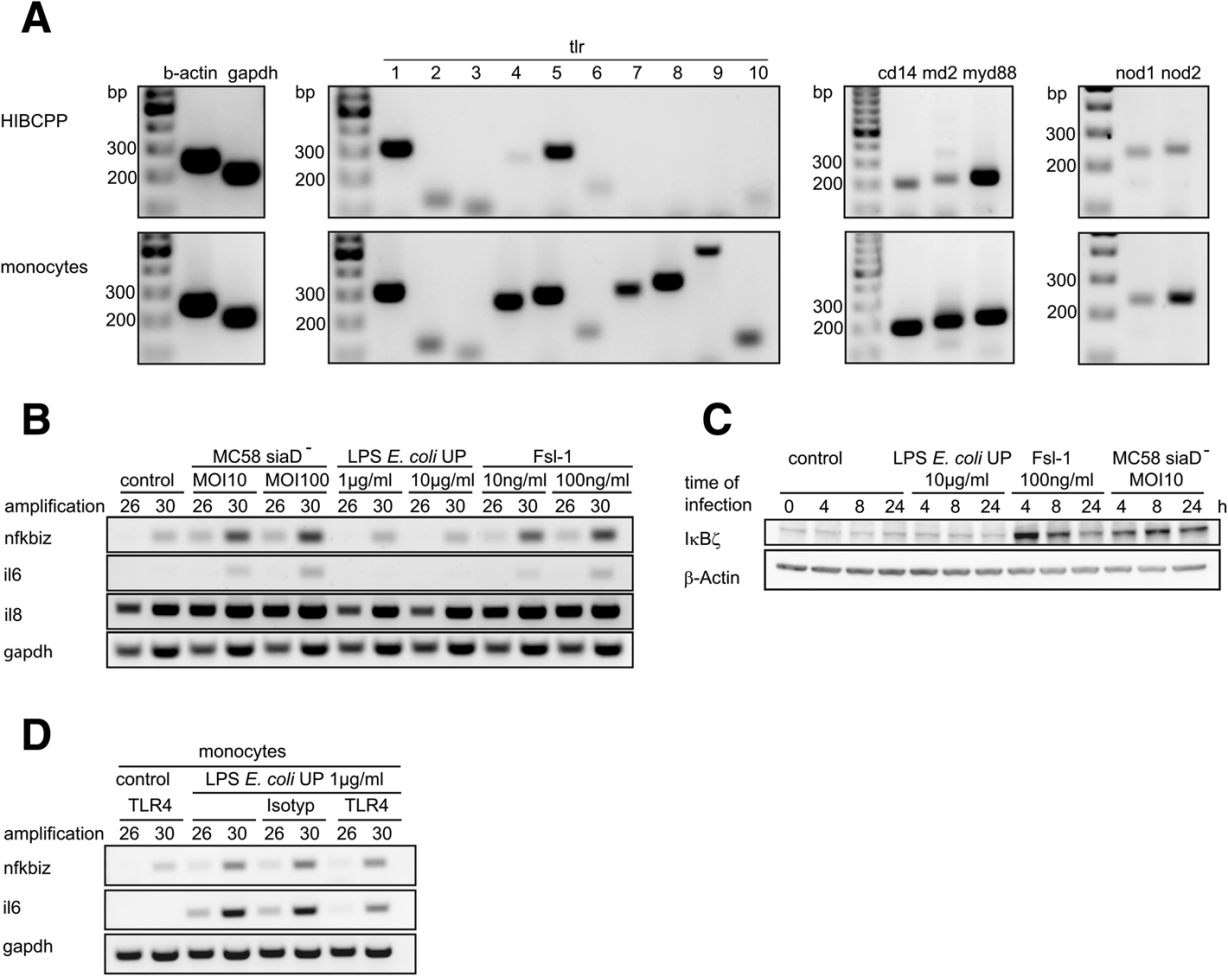
**TLR4 does not mediate gene induction caused by** ***Nm*** **. (A)** Expression profile of PRRs in HIBCPP cells. Qualitative RT-PCR was performed to determine the expression of the indicated PRRs, co-receptors, and adaptor molecules in HIBCPP cells (upper panel) and purified human monocytes (lower panel). Expression of β-actin and GAPDH was analyzed as control. The size of relevant marker nucleic acids is indicated. **(B)** Semi-quantitative RT-PCR was performed to measure activation of *nfkbiz*, *il6*, and *il8* after 4 h treatment with either *Nm* MC58siaD^−^, “ultrapure” LPS, or Fsl-1 at the indicated multiplicity of infection (MOI) or concentrations, respectively. Control experiments were performed in absence of a stimulus. PCR reactions were analyzed after the cycle numbers indicated at the top of the panels. **(C)** HIBCPP cells were challenged with 10 μg mL^−1^ “ultrapure” LPS or 100 ng mL^−1^ Fsl-1 for the indicated time. Expression of IκBζ protein was analyzed by immunoblot analysis. Detection of β-actin protein levels served as control. **(D)** Semi-quantitative RT-PCR was performed to determine activation of *nfkbiz* and *il6* in primary human monocytes after treatment with 1 μg mL^−1^ “ultrapure” LPS. Inhibition experiments were performed with an antibody against human TLR4 or with an isotype control, respectively.

Up-regulation of cytokines/chemokines as well as transcription factors like IκBζ in response to bacterial infection can be mediated by PRRs including TLRs. We were interested to investigate the receptors involved in signaling in response to neisserial infection of HIBCPP cells. To determine the expression profile of PRRs and described co-receptors in HIBCPP cells we performed qualitative RT-PCR analyses. As can be seen in Figure 5A expression of TLR1, TLR2, TLR3, TLR4, TLR5, TLR6, and TLR10 could be detected. HIBCPP cells also expressed the co-receptors CD14 and MD2, the adapter molecule MyD88, and the two intracellular PRRs Nod1 and Nod2. TLR7, TLR8, and TLR9 were not detected. All investigated transcripts could be demonstrated by analyzing control RNA isolated from purified human monocytes, but it should be noted that in some control samples TLR9 was not found (data not shown). Although the results of qualitative RT-PCR have to be judged carefully, it seemed that the transcript levels of several of the investigated factors expressed by HIBCPP cells (i.e., TLR4, TLR10, CD14, MD2, and Nod2) were rather low when compared to human monocytes (Figure 5A).

We used semiquantitative RT-PCR to analyze target gene expression in HIBCPP cells following stimulation with specific TLR agonists. A TLR4 agonist, “ultrapure” LPS, failed to induce *nfkbiz* and *il6* in HIBCPP cells after 4 h of stimulation (Figure 5B). Also, no induction of IκBζ protein expression was found by western blotting under these conditions (Figure 5C). Primary human monocytes were used as controls to prove functionality of applied stimuli. Stimulation of monocytes with the “ultrapure” LPS resulted in a strong *nfkbiz* and *il6* induction, which could be attenuated with a specific antibody against TLR4 (Figure 5D).

TLR2-mediated signal transduction can involve TLR2/TLR6 as well as TLR2/TLR1 receptor complexes [9]. Expression of *nfkbiz* and *il6* could be induced by the specific TLR2/TLR6 stimulus Fsl-1 in HIBCPP cells as well as human monocytes (Figure 5B and data not shown) and IκBζ protein was strongly expressed in HIBCPP cells after stimulation with Fsl-1 (Figure 5C).

**Figure 6:**
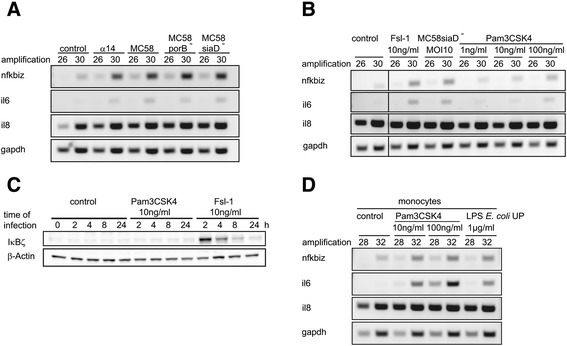
**The role of TLR2/TLR1 during** ***Nm*** **-mediated gene activation. (A)** Semi-quantitative RT-PCR was performed to measure activation of *nfkbiz*, *il6*, and *il8* after 4 h treatment with either α14, MC58, MC58porB^−^, or MC58siaD^−^ *Nm* strains at a multiplicity of infection (MOI) of 10, respectively. Control experiments were performed in absence of a stimulus. PCR reactions were analyzed after the cycle numbers indicated at the top of the panels. **(B)** Semi-quantitative RT-PCR was performed to measure activation of *nfkbiz*, *il6*, and *il8* after 4 h treatment with either Fsl-1, *Nm* MC58siaD^−^, or PAM3CSK4 at the indicated MOI or concentrations, respectively. Control experiments were performed in absence of a stimulus. PCR reactions were analyzed after the cycle numbers indicated at the top of the panels. **(C)** HIBCPP cells were challenged with 10 ng mL^−1^ PAM3CSK4 or Fsl-1 for the indicated time. Expression of IκBζ protein was analyzed by immunoblot analysis. Detection of β-actin protein levels served as control. **(D)** Semi-quantitative RT-PCR was performed to determine activation of *nfkbiz*, *il6*, or *il8* in primary human monocytes after treatment with the indicated amounts of PAM3CSK4 or “ultrapure” LPS. Control experiments were performed in absence of a stimulus. PCR reactions were analyzed after the cycle numbers indicated at the top of the panels.

The PorB protein of *Nm* is considered as a TLR2/TLR1 ligand [50]. Infection of HIBCPP cells with a PorB-deficient mutant of the MC58 strain for 4 h resulted in a stimulation of *nfkbiz* and *il6* comparable to the PorB-containing strains (Figure 6A). Furthermore, the synthetic TLR2/TLR1 ligand PAM3CSK4 caused only weak induction of *nfkbiz* and *il6* (Figure 6B). No expression of IκBζ protein was observed after treatment of HIBCPP cells with 10 ng mL^−1^ PAM3CSK4, whereas the same concentration of Fsl-1 caused a strong induction of IκBζ (Figure 6C). In contrast to HIBCPP cells, primary human monocytes displayed a strong activation of *nfkbiz* and *il6* after treatment with PAM3CSK4 (Figure 6D).

## Discussion

Only limited knowledge regarding the response of the CP to infection with pathogenic bacteria is available. Recently, employing the zoonotic agent *S. suis* and PCPEC, our laboratory had shown that porcine CP cells produce pro-inflammatory cytokines and chemokines *in vitro* when stimulated from the apical side [25]. In the current study, we used the inverted cell culture insert model of HIBCPP cells to investigate the impact of carrier and disease isolates of the human-specific bacterium *Nm* following infection from the physiologically-relevant basolateral side in a human model of the BCSFB. *Nm* has been shown to interact during infection with the CP epithelium [5,6], which constitutes the morphological correlate of the BCSFB [4].

The CSF is an immunologically privileged site due to the large absence of soluble PRRs, which recognize bacteria and enhance their uptake, as well as of components important for the adaptive immune system [51]. Therefore, the release of cytokines and chemokines from structures surrounding the CSF like the CP or from antigen presenting cells present in these structures plays a critical role during host defense and disease progress. Recognition and elimination of pathogens by those sentinel immune cells can cause further release of signaling molecules leading to activation of neutrophils and their infiltration into the CNS with severe consequences for the host, which is a pathological hallmark of bacterial meningitis [26,51]. Release of cytokines and chemokines for defense purposes after infection with the MC58 *Nm* strain has been described for human meningothelial meningioma cells and, based on transcriptome analyses, for human brain microvascular endothelial cells [52-55]. In agreement with these studies, we detected production of TNFα, IL8, and IL6 by HIBCPP cells after exposure to *Nm*. It should be noted that the amount of IL6 produced by HIBCPP cells is quite low, especially when compared to expression levels reached in meningothelial meningioma cells [54,55]. These differences can be possibly attributed to the distinct cell types used in the experiments and to the fact that HIBCPP cells do not necessarily mirror the amounts of IL6 produced by CP epithelial cells *in vivo*. Noteworthy, we observed induction of the *Zc3H12a* gene, whose product acts as an RNAse and can degrade mRNA encoding for IL6 [56], in HIBCPP cells after infection with the MC58 strain. Our data point to a less important role of the CP epithelium in IL6-mediated innate immune responses compared to meningothelial cells. Chemokines strongly induced by infected HIBCPP cells included CCL20 and CXCL1–3. Interestingly, stimulation of HIBCPP with enterovirus lead to the increase of CXCL1–3 expression as well [44]. These chemokines are expressed at sites of inflammation and have been described to attract neutrophils, but also monocytes [57]. This is in agreement with the observed transmigration of polymorphonuclear neutrophils and monocytes through HIBCPP layers after bacterial infection [58]. Along these lines, we found up-regulation of the gene encoding for ICAM-1, which serves as a ligand for leukocyte receptors [24], in HIBCPP after infection with *Nm*. Noteworthy, induced levels were also detected for G-CSF and GM-CSF, which are colony stimulating factors for the development of granulocytes and monocytes from stem cells, respectively [59].

We observed that expression of a capsule by the MC58 strain strongly reduced the number of genes regulated in HIBCPP cells, correlating with an attenuation of invasion into HIBCPP cells from the basolateral side as previously described [7]. A capsule-dependent influence on host cell invasion and regulation of gene expression by *Nm* has also been described previously [53,60,61]. Still, capsular polysaccharides of *Nm* can induce inflammatory responses via TLR2 and TLR4 [62]. The release of inflammatory mediators can be inhibited by interaction of the human host defense peptide LL-37 with capsular polysaccharides [63]. Noteworthy, LL-37 is not up-regulated in HIBCPP cells after infection with *Nm*, Fsl-1, or PAM3CSK4 as judged by the microarrays or semiquantitative RT-PCR (data not shown). In contrast, the microarray analyses indicated that strain MC58siaD^−^ specifically induced elevated levels of the chemokines CXCL5 and CXCL6, which also possess chemotactic activity for neutrophils [57], further supporting the induction of chemokines attracting neutrophils by *Nm*. Interestingly, most of the identified regulated chemokines (i.e., panGRO/CXCL1-3, CXCL5, CXCL6, and CXCL8) are ERL motif positive chemokines, which exhibit particular specificity for neutrophils [64]. Although pathogenic *Nm* strains are generally encapsulated, the effects observed with the capsule-deficient mutant may be relevant, since it has been discussed for *Nm* and *Streptococcus pneumoniae* that the capsule is down-regulated upon contact with host cells [65-67]. Further, some clinical evidence for capsular expression switching during meningococcal disease has been provided by the observation that meningococci isolated from the nasopharynx of patients display variable degrees of encapsulation; often, capsule-negative bacteria are found [68]. Interestingly, among the *Nm* strains investigated, the carrier isolate α14, which is incapable of capsule production [69], displayed the lowest levels of invasion and gene regulation, indicating a lack of factors required for invasion. The α14 strain has class I pili as MC58 (Dr. H. Claus, personal communication), but lacks the outer membrane opacity protein Opc as determined by using the Neisseria Multi Locus Sequence Typing website (http://pubmlst.org/neisseria/). Whereas the presence of pili could cause activation of certain host cell genes by α14, the lack of Opc provides an explanation for the decreased level of gene regulation compared to MC58siaD^−^, since Opc is exposed to host cell surfaces in the absence of the bacterial capsule [70].

Employing UV-inactivated bacteria we found that host gene activation was still caused by the inactivated *Nm* (Additional file 2: Figure S1). This result is not necessarily surprising, since activation of cytokines and chemokines has also been observed for heat-inactivated *Nm* in HEK293 cells expressing TLR2 or TLR4, respectively [71]. Activation of target genes has also been detected for UV-inactivated and heat-inactivated *S. suis* [72-74]. Additionally, we found ingestion of UV-inactivated *Nm* by HIBCPP cells (data not shown), which has also been observed during treatment of PCPEC with UV-inactivated *S. suis* [72]. Interestingly, employing the UV-inactivated bacteria, gene activation by MC58 and MC58siaD^−^ is reduced in relation to the α14 strain. These results suggest that additional inflammatory processes are involved when HIBCPP cells are infected with the living disease isolate MC58 and its acapsular mutant.

A few studies have been performed comparing the impact on host cells by pathogenic and apathogenic *Neisseria*. Fowler et al. [75] have shown that *Nm* induces higher levels of IL6 and a stronger down-regulation of the chemokine RANTES than the closely related apathogenic organism *Neisseria lactamica* (*N. lactamica*) in human meningothelial meningioma cells. In addition, *Nm*, but not *N. lactamica*, caused death of the host cells [75]. In a different study, transcriptome analysis showed down-regulation of host defense genes in 16HBE14 human bronchial epithelial cells after exposure to *Nm* relative to *N. lactamica* [76]. Interestingly, infection of a human endometrial epithelial cell line with invasive or non-invasive meningococcal isolates revealed that only invasive *Nm* caused a late repression of NF-κB activity, which lead to host cell apoptosis [77]. We observed a significant stronger activation of primary NF-κB target genes, i.e., *cxcl2*, *cxcl3*, and *nfkbiz*, which encodes the nuclear IκB protein IκBζ, by the more invasive disease isolate MC58 and its acapsular mutant compared to strain α14. In agreement with a function of the IκBζ pathway during infection of HIBCPP cells with *Nm*, IκBζ target genes were induced on RNA (*il6*) as well as on protein level (*il6*, *g-csf*, *gm-csf*). It should be noted that, at later time points of infection, expression levels of *nfkbiz* and *il6* caused by strains α14 and MC58 reached that of MC58siaD^−^, indicating that the possible maximum levels of gene activation are finally induced by all three intracellularly replicating strains and/or extracellular bacterial components present in the media after antibiotic treatment at 4 h. Although to our knowledge a role of IκBζ during meningococcal infection has not been reported up to now, IκBζ-induced expression of IL6 was described for human lung epithelial cells after treatment with the Gram-negative bacterium *Legionella pneumophila* [19]. A possible involvement of IκBζ during CNS disease is supported by the observation that the meningitis-causing yeast *Cryptococcus neoformans* up-regulates the *nfkbiz* gene in murine dendritic cells [78], and polymorphisms in *nfkbiz* are associated with invasive pneumococcal disease [79].

Signal transduction initiated by gram-negative pathogens involves the interaction of bacterial LPS with the TLR4 signaling complex [9]. The importance of TLR4 during infection with *Nm* is reflected by the close association of disease severity and inflammatory response with the level of LPS in plasma and CSF [3]. Both expression analysis and specific TLR4 stimulation indicated that TLR4 is only expressed at marginal levels in HIBCPP cells, which explains the lack of response of HIBCPP cells to “ultrapure” LPS. *Nm*-induced signal transduction in target cells can also be mediated by TLR9 or by TLR2, concerning the latter with an indication for an important role of the neisserial porin [50,71,80]. In this regard, the PorB protein of *Nm* has been shown to bind to TLR2 and to require TLR1 for signaling [50]. Since activation of *nfkbiz* and *il6* is not attenuated after infection with a PorB-deficient MC58 strain, signal transduction initiated by TLR2/TLR1 does not seem to play a major role in HIBCPP cells, at least for stimulation of the IκBζ-IL6 axis. This conclusion is supported by the observation that the synthetic TLR2/TLR1 ligand, PAM3CSK4, causes only weak activation of *nfkbiz* and *il6*. In contrast, stimulation with the TLR2/TLR6 ligand Fsl-1 leads to strong activation of *nfkbiz* and *il6* comparable to infection with MC58siaD^−^, suggesting that diacetylated lipopeptides binding to TLR2/TLR6 complexes are involved in gene regulation in HIBCPP cells by *Nm*.

Knowledge regarding the expression pattern of TLRs in the brain, especially in the CP, is, thus far, only available from studies in mice and rats [81,82] and little is known about their expression in the human brain. Nagyoszi et al. [46] demonstrated the expression of TLR2, TLR3, TLR4, and TLR6 on rat and human cerebral endothelial cells, and expression of several TLRs was analyzed in the human CNS using human microglia, astrocytes, and oligodendrocytes [83]. Although it is difficult to judge whether HIBCPP cells faithfully reflect the TLR expression pattern in the human brain, our data show that activation of TLR2/TLR6 can cause changes in gene expression levels correlating with those induced by *Nm*. Our observation that activation of host cell genes correlates with invasion levels of *Nm* also points to a possible role of endogenous PRRs, including NOD1 and NOD2, which are expressed in HIBCPP cells. Importantly, IκBζ has been shown to play an important role in NOD-like receptor ligand-mediated inflammation [16]. Furthermore, in mice, cytoplasmic LPS has been shown to activate the non-canonical inflammasome involving caspase-11, a process that is TLR4-independent [84,85]. A critical role of caspase-4, a human homolog of caspase-11, in endotoxin sensitivity has just recently been described in transgenic mice expressing human caspase-4 in its genomic context, and the *Shigella* OspC3 Effector has been shown to inhibit caspase-4 and to promote epithelial infection [86,87].

## Conclusions

In conclusion, our data show that *Nm* can induce the expression of cytokines and chemokines in CP epithelial cells involving activation of the IκBζ pathway, probably via TLR2/TLR6 or endogenous PRRs and cytosolic sensing of LPS/lipooligosaccharide. A better understanding of the mechanisms that underlie the host cell response during the course of bacterial meningitis will be useful in improving treatment of this disease.

## Additional files

Additional file 1: Table S1. Genes significantly regulated by MC58, the capsule-deficient mutant strain MC58siaD^−^ and the carrier isolate strain α14 with FC ≥1.5 FC ≤0.67 and a corresponding *P* value ≤0.001. Table S1 lists all genes which have been identified during the microarray analysis by comparison of untreated control cells with HIBCPP infected with strains α14, MC58, or the capsule-deficient mutant strain MC58siaD^−^, respectively. Additional file 2: Figure S1. Gene induction in HIBCPP cells caused by living and UV-inactivated *Nm*. HIBCPP cells were infected or stimulated for 4 h as indicated and the expression of *nfkbiz*, *il6*, *zc3h12a*, *il8*, and *gapdh* was documented by semi-quantitative RT-PCR. Control experiments were performed in absence of a stimulus or in case of inactivated *Nm* with addition of UV-irradiated cell medium. PCR reactions were analyzed after the cycle numbers indicated at the top of the panels.

## Abbreviations

BCSFB: Blood-cerebrospinal fluid barrier
CNS: Central nervous system
CP: Choroid plexus
CSF: Cerebrospinal fluid
FCS: Fetal calf serum
GO: Gene ontology
GSEA: Gene set enrichment analysis
HIBCPP: Human choroid plexus papilloma
IκB: Inhibitory κB
LPS: Lipopolysaccharide
*Nm*: *Neisseria meningitidis*
PAMP: Pathogen-associated molecular pattern
PBMC: Peripheral blood mononuclear cell
PPM: Proteose peptone medium
PRR: Pattern recognition receptor
qPCR: Quantitative real-time PCR
TEER: Transepithelial electrical resistance
TIR: Toll/Interleukin-1 receptor
TLR: Toll-like receptor
