# Supplementary material for: Neisseria meningitidis elicits a pro-inflammatory response involving IκBζ in a human blood-cerebrospinal fluid barrier model
Source: J Neuroinflammation. 2014 Sep 13;11:163. doi: 10.1186/s12974-014-0163-x (PMC4172843; doi:10.1186/s12974-014-0163-x)
Supplement: Additional file 1: Table S1. — Genes significantly regulated by MC58, the capsule-deficient mutant strain MC58siaD− and the carrier isolate strain α14 with FC ≥1.5 FC ≤0.67 and a corresponding P value ≤0.001. Table S1 lists all genes which have been identified during the microarray analysis by comparison of untreated control cells with HIBCPP infected with strains α14, MC58, or the capsule-deficient mutant strain MC58siaD−, respectively. [file 12974_2014_163_MOESM1_ESM.pdf]

Table S1

Genes significantly regulated by MC58, the capsule deficient mutant strain MC58siaD⁻ and the carrier isolate strain α14 with FC≥1.5 FC≤0.67 and a corresponding *P*-value ≤0.001. The Additional Table 1 lists all genes which have been identified during the microarray analysis by comparison of untreated control cells with HIBCPP infected with strains α14, MC58 or the capsule deficient mutant strain MC58siaD$^-$, respectively.

UniGene_ID	Gene Title	Gene Symbol	Foldchange<br>α14 vs. control	MC58 vs. control	MC58siaD$^-$ vs. control
Hs.164021	chemokine (C-X-C motif) ligand 6 (granulocyte chemotactic protein 2)	CXCL6			2,54
Hs.525607	tumor necrosis factor, alpha-induced protein 2	TNFAIP2			2,10
Hs.77274	plasminogen activator, urokinase	PLAU			2,05
Hs.436061	interferon regulatory factor 1	IRF1			1,98
Hs.25829	RAS, dexamethasone-induced 1	RASD1			1,93
Hs.522109	solute carrier family 6 (amino acid transporter), member 14	SLC6A14			1,91
Hs.62192	coagulation factor III (thromboplastin, tissue factor)	F3			1,88
Hs.171426	nuclear receptor coactivator 7	NCOA7			1,85
Hs.515415	inositol 1,4,5-trisphosphate 3-kinase C	ITPKC			1,85
Hs.466871	plasminogen activator, urokinase receptor	PLAUR			1,82
Hs.632267	syndecan 4	SDC4			1,81
Hs.89663	cytochrome P450, family 24, subfamily A, polypeptide 1	CYP24A1			1,78
Hs.124940	Rho family GTPase 1	RND1			1,76
Hs.489615	nicotinamide phosphoribosyl-transferase	NAMPT			1,74
Hs.432132	G0/G1switch 2	G0S2			1,72
Hs.497822	dual specificity phosphatase 10	DUSP10			1,70
Hs.723876	---				1,70
Hs.104879	serpin peptidase inhibitor, clade B (ovalbumin), member 9	SERPINB9			1,70
Hs.530381	pim-3 oncogene	PIM3			1,66
Hs.115263	epiregulin	EREG			1,65
Hs.602497	236982_at	---			1,65
Hs.287702	ADP-ribosylation factor-like 14	ARL14			1,65
Hs.98309	interleukin 23, alpha subunit p19	IL23A			1,64

UniGene_ID	Gene Title	Gene Symbol	Foldchange α14 vs. control	MC58 vs. control	MC58siaD⁻ vs. control
Hs.723465	238727_at				1,63
Hs.171596	EPH receptor A2	EPHA2			1,62
Hs.593014	UDP-glucose ceramide glucosyltrans-ferase	UGCG			1,59
Hs.654950					1,59
Hs.659056					1,58
Hs.283565	FOS-like antigen 1	FOSL1			1,58
Hs.475055	BCL2-interacting killer (apoptosis-inducing)	BIK			1,56
Hs.592304	ERO1-like (S. cerevisiae)	ERO1L			1,56
Hs.594569	---	---			1,55
Hs.487046	superoxide dismutase 2, mitochondrial	SOD2			1,55
Hs.591868	zinc finger and BTB domain containing 10	ZBTB10			1,55
Hs.512211					1,54
Hs.89714	chemokine (C-X-C motif) ligand 5	CXCL5			1,54
Hs.446315	family with sequence similarity 107, member B	FAM107B			1,53
Hs.477866	procollagen-lysine, 2-oxoglutarate 5-dioxygenase 2	PLOD2			1,53
Hs.591569	SERTA domain containing 2	SERTAD2			1,53
Hs.1027	Ras-related associated with diabetes	RRAD			1,53
Hs.193516	B-cell CLL/lymphoma 10	BCL10			1,53
Hs.511899	endothelin 1	EDN1	1,51	(1,47)	1,53
Hs.31210	B-cell CLL/lymphoma 3	BCL3			1,52
Hs.636188					1,52
Hs.506381	FYVE, RhoGEF and PH domain containing 6	FGD6			1,52
Hs.376289	zinc finger CCCH-type containing 12C	ZC3H12C			1,51
Hs.255935	B-cell translocation gene 1, anti-proliferative	BTG1			1,51
Hs.534293	serpin peptidase inhibitor, clade A (alpha-1 antiproteinase, antitrypsin), member 3	SERPINA3			1,51
Hs.534313	early growth response 3	EGR3			1,50
Hs.621704					1,50
Hs.458276	nuclear factor of kappa light polypeptide gene enhancer in B-cells inhibitor, epsilon	NFKBIE			1,50
Hs.131226	BCL2/adenovirus E1B 19kDa interacting protein 3-like	BNIP3L			1,50
Hs.410037	connective tissue growth factor	CTGF			0,65
Hs.503345	aquaporin 11	AQP11			0,65

UniGene_ID	Gene Title	Gene Symbol	Foldchange<br>α14 vs. control	MC58 vs. control	MC58siaD⁻ vs. control
Hs.78944	regulator of G-protein signaling 2, 24kDa	RGS2			0,64
Hs.710157	ovo-like 2 (Drosophila)	OVOL2			0,64
Hs.408542	protein kinase domain containing, cytoplasmic homolog (mouse)	PKDCC			0,63
Hs.525091	target of EGR1, member 1 (nuclear)	TOE1			0,63
Hs.180919	inhibitor of DNA binding 2, dominant negative helix-loop-helix protein	ID2			0,62
Hs.319171	Nuclear factor of kappa light polypeptide gene enhancer in B-cells inhibitor, zeta	NFKBIZ		2,36	4,18
Hs.196384	prostaglandin-endoperoxide synthase 2 (prostaglandin G/H synthase and cyclooxygenase)	PTGS2		1,67	3,33
Hs.376208	Lymphotoxin beta (TNF superfamily, member 3)	LTB		1,68	2,85
Hs.656294	zinc finger CCCH-type containing 12A	ZC3H12A		1,92	2,75
Hs.241570	tumor necrosis factor (TNF superfamily, member 2)	TNF		1,73	2,67
Hs.591849	chromosome 8 open reading frame 4	C8orf4		1,68	2,41
Hs.127799	baculoviral IAP repeat-containing 3	BIRC3		1,86	2,40
Hs.2250	leukemia inhibitory factor (cholinergic differentiation factor)	LIF		1,52	2,05
Hs.656630	230333_at	---		1,80	2,05
Hs.432453	mitogen-activated protein kinase kinase kinase 8	MAP3K8		1,83	1,99
Hs.2128	dual specificity phosphatase 5	DUSP5		1,53	1,95
Hs.73853	bone morphogenetic protein 2	BMP2		1,53	1,87
Hs.642842	salvador homolog 1 (Drosophila)	SAV1		1,52	1,74
Hs.371240	A kinase (PRKA) anchor protein 12	AKAP12		1,60	1,74
Hs.67928	E74-like factor 3 (ets domain transcription factor, epithelial-specific)	ELF3		1,58	1,69
Hs.652855		---		1,55	1,60
Hs.374950	metallothionein 1X	MT1X		1,61	1,60
Hs.146339	protein phosphatase 2 (formerly 2A), regulatory subunit B, alpha isoform	PPP2R2A		1,52	1,54
Hs.89404	msh homeobox 2	MSX2		0,65	0,66
Hs.708635				0,66	0,64
Hs.75498	chemokine (C-C motif) ligand 20	CCL20	3,72	5,49	12,82
Hs.441047	adrenomedullin	ADM	10,77	12,01	11,13
Hs.9613	angiopoietin-like 4	ANGPTL4	8,06	8,82	9,60
Hs.75765	chemokine (C-X-C motif) ligand 2	CXCL2	1,86	3,34	7,51

			Foldchange		
UniGene_ID	Gene Title	Gene Symbol	α14 vs. control	MC58 vs. control	MC58siaD[-] vs. control
Hs.419240	solute carrier family 2 (facilitated glucose transporter), member 3	SLC2A3	5,61	6,01	5,94
Hs.89690	chemokine (C-X-C motif) ligand 3	CXCL3	1,55	2,62	5,09
Hs.712599	DNA-damage-inducible transcript 4	DDIT4	4,04	4,43	4,42
Hs.674926			3,52	3,99	4,27
Hs.195471	6-phosphofructo-2-kinase/fructose-2,6-biphosphatase 3	PFKFB3	4,02	4,11	4,23
Hs.706124	chromosome 7 open reading frame 68 Hypoxia-inducible lipid droplet-associated protein	C7orf68	3,26	3,61	4,08
Hs.724593	arrestin domain containing 3	ARRDC3	3,50	4,05	4,08
Hs.643447	intercellular adhesion molecule 1	ICAM1	1,67	2,18	3,92
Hs.624	interleukin 8	IL8	1,52	2,16	3,82
Hs.516664	ephrin-A1	EFNA1	2,91	3,09	3,47
Hs.211600	tumor necrosis factor, alpha-induced protein 3	TNFAIP3	1,59	2,13	3,44
Hs.81328	nuclear factor of kappa light polypeptide gene enhancer in B-cells inhibitor, alpha	NFKBIA	1,58	2,18	3,38
Hs.506076	protein tyrosine phosphatase, receptor type, R	PTPRR	3,21	3,32	3,32
Hs.135507	egl nine homolog 3 (C. elegans)	EGLN3	3,26	3,23	3,27
Hs.508154	ankyrin repeat domain 37	ANKRD37	3,07	3,12	3,07
Hs.450230	insulin-like growth factor binding protein 3	IGFBP3	2,57	2,75	3,07
Hs.642938	insulin-like growth factor binding protein 1	IGFBP1	2,22	2,45	3,06
Hs.605445	ERBB receptor feedback inhibitor 1	ERRFI1	2,13	2,23	2,75
Hs.372914	N-myc downstream regulated gene 1	NDRG1	2,74	2,80	2,74
Hs.592112	potassium channel tetramerisation domain containing 11	KCTD11	2,49	2,61	2,66
Hs.588854	fucosyltransferase 11 (alpha (1,3) fucosyltransferase)	FUT11	2,58	2,64	2,57
Hs.591588			2,35	2,44	2,50
Hs.591785	immediate early response 3	IER3	1,74	2,01	2,41
Hs.557425	jumonji domain containing 1A	JMJD1A	2,38	2,47	2,38
Hs.1407	endothelin 2	EDN2	2,36	2,66	2,36
Hs.594942			1,84	2,06	2,33
Hs.517617	v-maf musculoaponeurotic fibrosarcoma oncogene homolog F (avian)	MAFF	1,69	1,85	2,31
Hs.534052	zinc finger protein 36, C3H type, homolog (mouse)	ZFP36	1,76	1,91	2,27

UniGene_ID	Gene Title	Gene Symbol	Foldchange<br>α14 vs. control	 MC58 vs. control	 MC58siaD⁻ vs. control
Hs.563344	nuclear receptor subfamily 4, group A, member 2	NR4A2	1,81	1,74	2,26
Hs.476217	6-phosphofructo-2-kinase/fructose-2,6-biphosphatase 4	PFKFB4	2,27	2,22	2,22
Hs.79334	nuclear factor, interleukin 3 regulated	NFIL3	2,28	2,21	2,22
Hs.165258	235739_at		1,81	1,66	2,22
Hs.602328			2,10	2,11	2,09
Hs.7089	insulin induced gene 2	INSIG2	1,91	2,00	2,09
Hs.97270	family with sequence similarity 13, member A1	FAM13A1	2,12	2,14	2,07
Hs.474935	sema domain, immunoglobulin domain (Ig), transmembrane domain (TM) and short cytoplasmic domain, (semaphorin) 4B	SEMA4B	1,92	1,99	2,07
Hs.4055	Kruppel-like factor 6	KLF6	1,58	1,77	2,00
Hs.616962	growth differentiation factor 15	GDF15	1,56	1,70	1,99
Hs.501023	MAX interactor 1	MXI1	2,10	2,13	1,98
Hs.25647	v-fos FBJ murine osteosarcoma viral oncogene homolog	FOS	2,33	2,09	1,96
Hs.235782	similar to Solute carrier organic anion transporter family, member 4A1 /// solute carrier organic anion transporter family, member 4A1	LOC100134295 /// SLCO4A1	1,64	1,74	1,95
Hs.599725			1,83	2,02	1,93
Hs.477547	transmembrane and coiled-coil domain family 1	TMCC1	1,79	1,92	1,86
Hs.75969	proline-rich nuclear receptor coactivator 1	PNRC1	1,61	1,66	1,83
Hs.458513	protein phosphatase 1, regulatory (inhibitor) subunit 3B	PPP1R3B	1,87	1,94	1,78
Hs.500761	solute carrier family 16, member 3 (monocarboxylic acid transporter 4)	SLC16A3	1,55	1,60	1,72
Hs.511915	enolase 2 (gamma, neuronal)	ENO2	1,64	1,74	1,71
Hs.302742	mitochondrial ribosomal protein S6	MRPS6	1,55	1,65	1,69
Hs.515032	MAP kinase interacting serine/threonine kinase 2	MKNK2	1,65	1,68	1,68
Hs.485892	zinc finger protein 292	ZNF292	1,67	1,74	1,68
Hs.724499	cyclin G2	CCNG2	1,61	1,66	1,68
Hs.205627	rearranged L-myc fusion	RLF	1,57	1,68	1,67
Hs.473721	solute carrier family 2 (facilitated glucose transporter), member 1	SLC2A1	1,58	1,62	1,67
Hs.724498	TCDD-inducible poly(ADP-ribose) polymerase	TIPARP	1,62	1,78	1,67
Hs.82071	Cbp/p300-interacting transactivator, with Glu/Asp-rich carboxy-terminal domain, 2	CITED2	1,97	1,90	1,66

UniGene_ID	Gene Title	Gene Symbol	Foldchange<br>α14 vs. control	 MC58 vs. control	 MC58siaD⁻ vs. control
Hs.591650	zinc finger protein 654	ZNF654	1,61	1,62	1,62
Hs.470633	pyruvate dehydrogenase kinase, isozyme 1	PDK1	1,53	1,60	1,60
Hs.171825	basic helix-loop-helix domain containing, class B, 2	BHLHB2	1,57	1,61	1,58
Hs.500047	procollagen-proline, 2-oxoglutarate 4-dioxygenase (proline 4-hydroxylase), alpha polypeptide I	P4HA1	1,51	1,53	1,56
Hs.266175	phosphoprotein associated with glycosphingolipid microdomains 1	PAG1	1,57	1,58	1,55
Hs.30561	RAD54 homolog B (S. cerevisiae)	RAD54B	1,57	1,55	1,53
Hs.3416	adipose differentiation-related protein	ADFP	1,52	1,55	1,51
Hs.513667	Nucleolar protein 3 (apoptosis repressor with CARD domain)	NOL3	1,51	1,56	1,51
Hs.469254	lysine-rich coiled-coil 1	KRCC1	(1,39)	1,54	(1,48)
Hs.153648	protein tyrosine phosphatase, receptor type, f polypeptide (PTPRF), interacting protein (liprin), alpha4	PPFIA4	(1,46)	1,51	(1,46)
Hs.98370	cytochrome P450, family 2, subfamily S, polypeptide 1	CYP2S1	(1,49)	1,51	(1,44)
Hs.724431	thioredoxin interacting protein	TXNIP	0,63	0,61	0,59
Hs.608486			0,54	0,54	0,54
