# Supplementary material for: Neisseria meningitidis elicits a pro-inflammatory response involving IκBζ in a human blood-cerebrospinal fluid barrier model
Source: J Neuroinflammation. 2014 Sep 13;11:163. doi: 10.1186/s12974-014-0163-x (PMC4172843; doi:10.1186/s12974-014-0163-x)
Supplement: Additional file 2: Figure S1. — Gene induction in HIBCPP cells caused by living and UV-inactivated Nm. HIBCPP cells were infected or stimulated for 4 h as indicated and the expression of nfkbiz, il6, zc3h12a, il8, and gapdh was documented by semi-quantitative RT-PCR. Control experiments were performed in absence of a stimulus or in case of inactivated Nm with addition of UV-irradiated cell medium. PCR reactions were analyzed after the cycle numbers indicated at the top of the panels. [file 12974_2014_163_MOESM2_ESM.pdf]

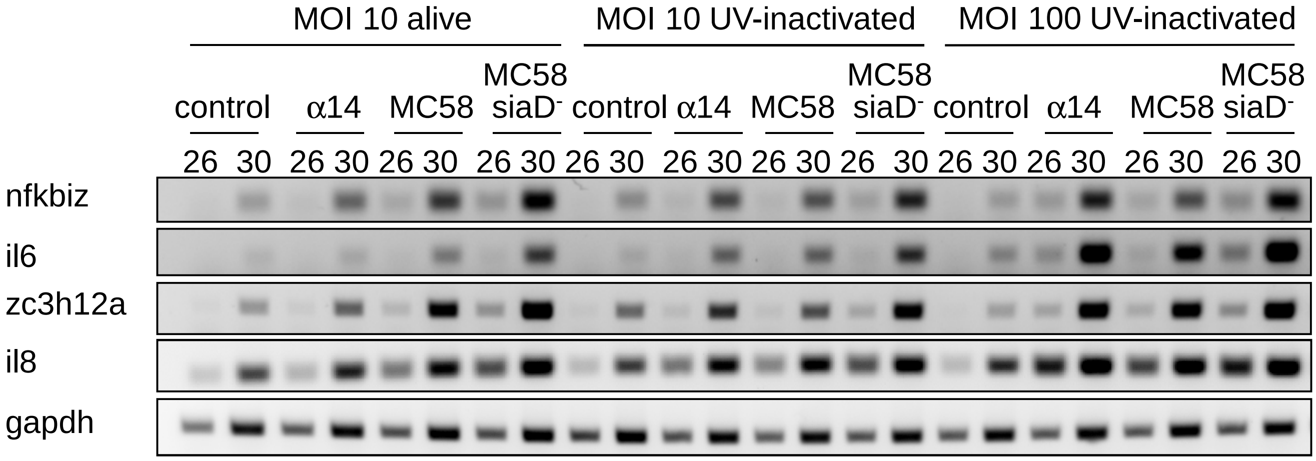

Figure S1

Gene induction in HIBCPP cells caused by living and UV-inactivated *Nm*. HIBCPP cells were infected or stimulated for 4 h as indicated and the expression of *nfkbiz, il6, zc3h12a, il8* and *gapdh* was documented by semi-quantitative RT-PCR. Control experiments were performed in absence of a stimulus or in case of inactivated *Nm* with addition of UV-irradiated cell medium. PCR reactions were analyzed after the cycle numbers indicated at the top of the panels.
